# Epitaxial Growth of SrTiO_3_ Films on Cube-Textured Cu-Clad Substrates by PLD at Low Temperature Under Reducing Atmosphere

**DOI:** 10.1186/s11671-017-1997-9

**Published:** 2017-03-28

**Authors:** J. A. Padilla, E. Xuriguera, L. Rodríguez, A. Vannozzi, M. Segarra, G. Celentano, M. Varela

**Affiliations:** 1 IN2UB, DIOPMA, Department of Materials Science and Physical Chemistry, Universitat de Barcelona, Martí i Franquès 1, 08028 Barcelona, Spain; 2 La Farga Lacambra SAU, Ctra. C17z Km. 73, 5, 08508 Les Masies de Voltregà, Barcelona, Spain; 3 Department of Applied Physics and Optics, Universitat de Barcelona, C/Martí i Franquès 1, 08028 Barcelona, Spain; 4 Superconductivity Laboratory, ENEA Frascati Research Centre, Via Enrico Fermi 45, I-00044 Frascati, Rome, Italy; 5 Catalan Institute of Nanoscience and Nanotechnology (ICN2), CSIC and The Barcelona Institute of Science and Technology, Campus UAB, 08193 Bellaterra, Spain

**Keywords:** Epitaxy, Thin film texture, High-temperature superconductivity, Pulsed laser deposition, Electron diffraction, X-ray diffraction

## Abstract

The growth of epitaxial {001}<100> SrTiO_3_ (STO) on low-cost cube-textured Cu-based clad substrate at low temperature was carried out by means of pulsed laser deposition (PLD). STO film was deposited in one step under a reducing atmosphere (5% H_2_ and 95% Ar mixture) to prevent the oxidation of the metal surface. The optimization of PLD parameters leads to a sharpest biaxial texture at a temperature as low as 500 °C and a thickness of 500 nm with a (100) STO layer. The upper limit of highly textured STO thickness was also investigated. The maximum thickness which retains the best quality {001}<100> texture is 800 nm, since the texture is preserved not only through the layer but also on the surface. Atomic force microscopy (AFM) and scanning electron microscopy (SEM) measurements showed that STO films are continuous, dense, and smooth with very low roughness (between 5 and 7 nm). This paper describes the development of STO layer by means of PLD in absence of oxygen throughout the process, suggesting an alternative and effective method for growing highly {001}<100> textured STO layer on low-cost metal substrates.

## Background

The close relationship between mechanical, structural, and electronic properties of perovskite-based oxides makes them extremely useful [1]. Strontium titanate (SrTiO_3_ (STO)) is a well-known and studied oxide with perovskite structure [2]. In fact, it is widely used as a common substrate for epitaxial growth of functional oxide systems, in most cases in the form of single crystals cut in a particular crystal plane [3], in different fields such as superconductors [4, 5], photovoltaic materials [6–8], thermoelectrics [9], and semiconductors [10, 11]. Thin films of STO can be deposited on a large variety of substrates, such as ceramics [12], semiconductors [13], or metal alloys [14], and can be grown epitaxially by both chemical [15] and physical methods [2].

One of the most effective methods for growing an epitaxial STO film is the pulsed laser deposition (PLD) [16, 17]. In order to grow layers of oxides with suitable stoichiometry, an oxidizing atmosphere with an oxygen pressure (pO_2_) above 10^−4^ mbar is required [12]. When STO is deposited on semiconductor or metallic substrates, the growth at high temperature under an oxygen atmosphere can produce the oxidation of the substrate surface. To avoid the undesirable oxidation of metal surface that can inhibit epitaxial growth of the STO film, only a few authors have reported the usage of a reducing atmosphere in the case of growth of CeO_2_ and STO layers on Ni alloys by PLD at high temperature [14, 18]. They proposed a two-step growing process at high temperatures: a first step in a reducing atmosphere until tens of nanometers of thickness and a second step in an oxidizing atmosphere to the final thickness [14, 18].

There is a great interest in growing biaxially textured STO on metallic substrates. STO layers can be used as a diffusion barrier of Cu^2+^ and O^2−^ [18, 19]. The standard thickness for a barrier layer is between 150 and 300 nm [20].

Nickel is commonly used as a base metal for electrode capacitors [11] and metallic substrate in coated conductor (CC) applications [21]. One of the problems with the use of pure nickel for CC applications is its ferromagnetism with a Curie temperature of 627 K and a saturation magnetization of 57.5 emu/g at *T* = 0 [22]. The ferromagnetism complicates the design of high-field magnets for critical applications and Ni-based tapes in alternating current applications which run the risk of increased energy loss, due to hysteretic loss in the magnetic material [22]. It is clearly desirable to develop suitable alloys with reduced ferromagnetism that can also be successfully biaxially textured [22]. The use of copper-based substrates is an attractive solution for these problems, particularly because Cu, since it is not ferromagnetic, does not have Curie temperature and also because it has relatively low cost of high-purity raw material compared to Ni [23–25]. The use of Ni-electroplated Cu tape to prevent the oxidation of Cu is not a problem because the Ni-electroplated Cu tape lost its ferromagnetism after the heat treatment; this is because the Ni atoms in the thin Ni-electroplated layer are easily diffused into the Cu tape, and the Ni-electroplated Cu tape is changed to the paramagnetic dilute Ni-alloyed Cu tape [26].

In the following study, a cube-textured copper-clad substrate has been proposed to grow (001) STO films. This substrate is a commercial clad supplied by Tanaka Kikinzoku Kogyo K.K. with low roughness and excellent mechanical properties. This clad is based on a rolling-assisted biaxially textured substrate (RABiTS) of cube-textured copper (30 μm) with a thin electroplated cube-textured nickel layer on it with a thickness of 1 μm, bonded to a stainless steel (SS) tape (80 μm) by surface activated bonding (SAB) technique [24, 27]. The detailed fabrication process has been described in a previous work [24]. The main role of the nickel layer is to act as a barrier layer for copper diffusion and prevent the oxidation of the copper tape [27].

The aim of this work is to present the development and optimization of the maximum achievable thickness of highly {001}<100> textured STO films on a low-cost cube-textured Cu-clad substrate, by means of PLD in one step at low temperature, using reducing atmosphere (5% H_2_ and 95% Ar mixture) to prevent the oxidation of metallic substrate during all the process, not only at the beginning.

## Methods

STO thin films were deposited using a PLD system equipped with a KrF excimer laser (248 nm, *Lambda Physik LPX 210Pro*). The STO target used is polycrystalline and stoichiometric with 98% of density. Substrates were glued onto the heater with silver paste to ensure both a good thermal contact and mechanical fixation. The substrate’s temperature was measured using a thermocouple embedded in the heater. The chamber was evacuated to a base pressure of 1 × 10^−5^ mbar, and then, it was refilled to working pressure with a mixture of 5% H_2_ and 95% Ar. The target-to-substrate distance was 50 mm, and the fluency (pulsed laser energy density) was between 1.5 and 2 J/cm^2^. Laser frequency was varied between 2 and 10 Hz.

Crystallographic structure of the layers was characterized by X-ray diffraction (XRD; Philips MRD), including *θ–*2*θ* scan, *ω*-scan, *ϕ*-scan, and pole figure to analyze the macrotexture since XRD gives average information about the texture over a large scale and deep layer. Surface’s morphology and microstructural analysis were characterized by scanning electron microscopy (SEM; FEI Quanta-200) and surface roughness by atomic force microscopy (AFM; Multimode 8 with Nanoscope® V). The microtexture of STO layers was analyzed by automated electron backscattering diffraction (EBSD; TSL OIM) [28], since EBSD in SEM provides a sample population of orientation measurements which can be linked individually to their location within a specimen (local and surface texture). STO film composition was estimated by X-ray photoelectron spectroscopy (XPS; PHI 5500 Multitechnique System).

## Results and Discussion

Surface quality of the substrate affects the epitaxy and integrity of layers deposited on top [29]. For this reason, it is mandatory to assure the quality of the Cu-clad surface, so the initial texture of the samples was characterized by EBSD and XRD. EBSD analysis reveal an excellent cube texture with a fraction of oriented area exceeding 99% with 12° tolerance angle (the maximum deviation angle for a certain orientation) and a small amount of twin boundaries of 1%. The full width at half maximum (FWHM) measured of *ω*-scans at the (002) peak was 7° and 9.5° in rolling (RD) and transverse direction (TD), respectively, and the FWHM of *ϕ*-scan at *ψ* = 54.7° of (111) peak was 7.1°. Therefore, the Cu-clad substrate shows a strong and good cube texture [29], very suitable for coated conductor, for example.

SEM analysis shows dense and smooth surface, except for some holes corresponding to cube twins of copper. Roughness is frequently measured and reported as a root-mean-square roughness (RMS or *R*_q_), where the data comes from a profile. For a three-dimensional surface, the root-mean-square height of the surface (*S*_q_) is calculated by including all surface heights from the reference plane [30]; so in this case, the roughness obtained is more representative. By AFM, the *S*_q_ value for the substrate in an area of 25 × 25 μm^2^ is 17 nm, a proper roughness for some applications [29].

During any PLD, factors such as type of atmosphere, chamber pressure, laser frequency, and temperature affect film growth [12, 31, 32]. The temperature was set up to 700 °C to evaluate the type of growth using a reducing atmosphere, a mixture of 5% H_2_ and 95% Ar, during the process [2]. The pressure values studied were 4 × 10^−4^ and 4 × 10^−3^ mbar, and the laser frequency values studied were 2 and 10 Hz.

**Fig. 1:**
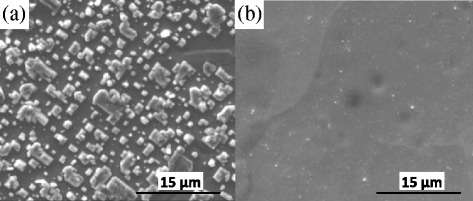
SEM images of STO films deposited at 700 °C with laser frequency and chamber pressure of **a** 2 Hz and 10^−3^ mbar and **b** 10 Hz and 10^−4^ mbar, respectively

In the case of a laser frequency of 2 Hz and a chamber pressure range between 10^−4^ and 10^−3^ mbar deposited at 700 °C, on the surface the formation of isolated structures can be observed (Fig. 1a). With a laser frequency of 10 Hz and a chamber pressure of 10^−3^ mbar deposited at 700 °C, the size of these crystals are much smaller as in the case of 2 Hz. Nevertheless, with a laser frequency of 10 Hz and a pressure of 4 × 10^−4^ mbar deposited at 700 °C (Fig. 1b), the surface show that (100) STO layers are continuous, dense, and smooth and show some defects with the aspect of cube twins. These defects seem to be the same as those observed in the surface of the tape without STO layer. This indicates that the STO film grows properly and reproduces the surface of the substrate.

The analysis by XRD of the STO sample deposited at a laser frequency of 10 Hz, a chamber pressure of 4 × 10^-4^ mbar, and a temperature of 700 °C shows a random orientation, even though the growth is very homogeneous with full coverage of the substrate. It is well-known that the temperature determines the mobility of deposited atoms on the surface and crystallization rate [12]. Because of this fact, different deposits have been performed from 300 to 850 °C with the laser frequency and the chamber pressure at 10 Hz and 4 × 10^−4^ mbar, respectively, attempting to obtain an epitaxial (100) STO layer with the sharpest {001}<100> texture.

For a previous determination of the temperature’s effect on epitaxial growth of STO layer, the orientation Lotgering factor of (200) STO peak from the *θ*–2*θ* scan (*f*_*L*(200)_) (Eq. 1) was used [5]. The *f*_*L*_, based on Lotgering orientation concepts [33], is calculated with the intensity of certain diffraction peaks in a *θ*–2*θ* scan, so the texture information obtained is qualitative. Diffraction peaks of 20° < 2*θ* < 50° were used for Eq. 1. The *f*_*L*(200)_ varies from 1 for perfectly {001} oriented to a low value greater than 0, corresponding to a randomly oriented sample (powder pattern) [11].

1 $$ {f}_{L(h00)}=\frac{{\displaystyle \sum }{I}_{h00}}{{\displaystyle \sum }{I}_{h00}+{\displaystyle \sum }{I}_{h kl}} $$

**Fig. 2:**
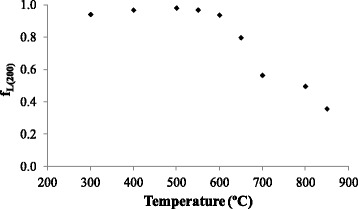
*f*_*L*(200)_ for STO films obtained between 200 and 850 °C at laser frequency and chamber pressure of 10 Hz and 4 × 10^−4^ mbar, respectively

Lotgering factor is a good qualitative measure of the out-of-plane (perpendicular to the sample plane) preferred orientation, but not yet the full texture (also in-plane) of the STO film. As can be seen in Fig. 2, in the range of 300 to 600 °C, the *f*_*L*(200)_ value is higher than 0.9. Therefore, {001} texture may be obtained in this temperature range [5]. Above 600 °C, the value of *f*_*L*(200)_ decreases significantly, indicating a lack of {001} texture.

**Fig. 3:**
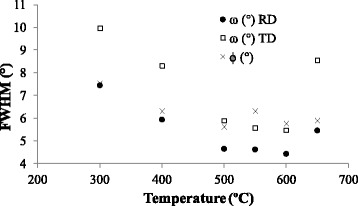
FWHM of (200) RD and TD of *ω*-scans and (111) *ϕ*-scans for the (100) STO films deposited between 300 and 650 °C at laser frequency and chamber pressure of 10 Hz and 4 × 10^−4^ mbar, respectively

To evaluate and quantify the quality of *c*-axis {001}<100> texture, FWHM is measured from *ω*-scans around RD and TD of (002) peak and *ϕ*-scan at *ψ* = 54.74° of (111) STO peak. For applications with a required sharp {001}<100> texture, FWHM values have to be as low as possible. In most cases, between 5° and 7° is considered a sharp texture [34]. As depicted in Fig. 3, the lowest values of FWHM are located between 500 and 600 °C. Below 500 °C, the samples obtained present {001}<100> texture, but the quality decreases since FWHM from *ω*-scans and *ϕ*-scan clearly increase. The values at 650 °C show the loss of texture at high temperatures, as inferred from the reduction of the preferential orientation in Fig. 2.

**Fig. 4:**
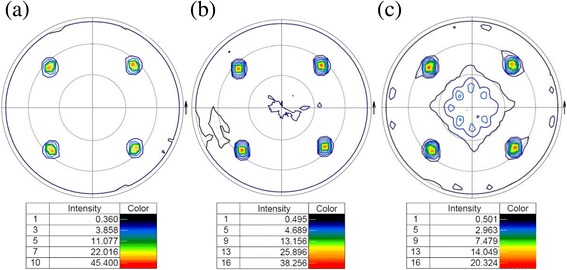
X-ray (111) pole figure in root scale of STO films deposited at laser frequency and chamber pressure of 10 Hz and 10^−4^ mbar, respectively, on Cu-clad substrate at temperatures of **a** 500 °C, **b** 550 °C, and **c** 600 °C

To determine if any secondary texture is present, the (111) pole figure is obtained by XRD for samples deposited at 500, 550, and 600 °C. A detailed analysis of pole figures indicates that the sample at 600 °C shows twins and another secondary texture with very low intensity (Fig. 4c) [35]. Furthermore, as can be observed in Fig. 4a, b, the samples at 500 and 550 °C show a very sharp {001}<100> texture without other orientations. The sample at 500 °C (Fig. 4a) is slightly better than the sample at 550 °C (Fig. 4b), since the normalized intensity is higher at 500 °C.

**Fig. 5:**
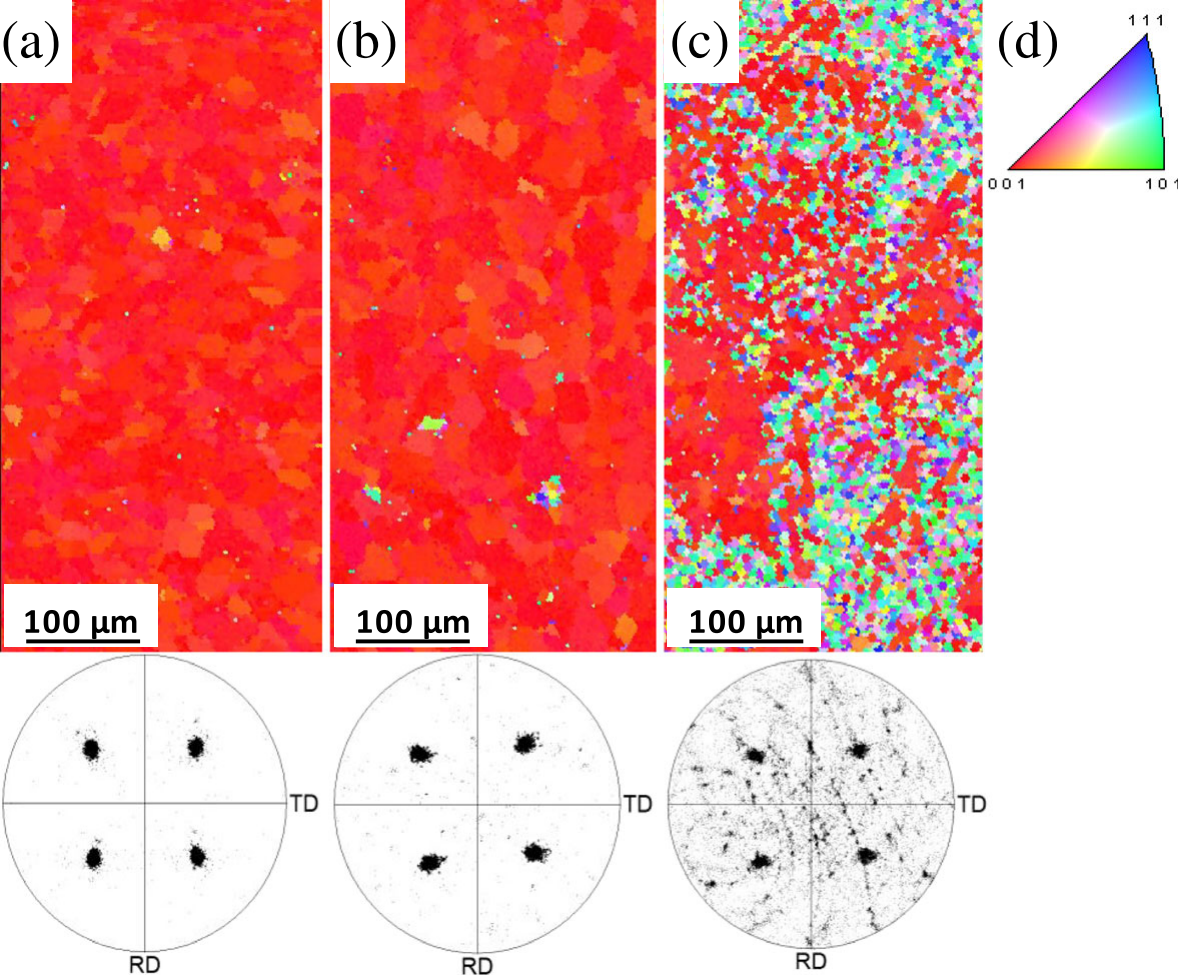
EBSD maps and the corresponding (111) pole figure of STO films grown at a chamber pressure, laser frequency, and substrate temperature of 10^−4^ mbar, 10 Hz, and 500 °C, respectively, with a thickness of **a** 500 nm, **b** 800 nm, **c** 1000 nm, and **d** color key for crystal orientation map

The microtexture of the sample at 500 °C (Fig. 5a) shows a surface fraction of {001}<100> texture of 99.5% with 12° tolerance angle that corresponds to a very strong texture. Therefore, an excellent {001}<100> texture is confirmed from both surface and through the entire layer. In this case, the growth rate is about 0.3 nm/s when the temperature of the deposition is 500 °C, and so the thickness of STO deposited at 500 °C is around 500 nm. At 500 °C, an excellent crystallinity and {001}<100> texture have been obtained, even at the low pressure of 10^−4^ mbar [2, 36]. It is widely known that STO layers can be used as a diffusion barrier of Cu^2+^ and O^2−^ in some applications [18, 19]; besides, its efficiency increases as the thickness of the STO layer increases. For this purpose, STO films were deposited with different thicknesses at optimal conditions of chamber pressure, laser frequency, and substrate temperature of 10^−4^ mbar, 10 Hz, and 500 °C, respectively.

The values of FWHM of *ω*-scans around RD and TD of (002) peak and *ϕ*-scan at *ψ* = 54.74° of (111) STO peak were obtained by XRD and are summarized in Table 1. The quality of {001}<100> texture is excellent in all the cases, regardless of the thickness. The biaxial-texture improvement of the textured films, with respect to the metal biaxial-textured substrates, is an effect that has already been observed [34].

**Table 1:** FWHM of *ω*-scans RD and TD of (002) peak and *ϕ*-scan at *ψ* = 54.74° of (111) peak for (100) STO films deposited at a chamber pressure, laser frequency, and substrate temperature of 10^−4^ mbar, 10 Hz, and 500 °C, respectively, with different thickness

STO thickness (nm)	FWHM (°)		
	*ω*-scan		*ϕ*-scan
	RD	TD	
500	4.6	5.9	5.6
800	4.5	6.2	5.3
1000	4.8	6.8	5.8

Figure 5 shows the EBSD maps and (111) pole figure of the obtained films with different thicknesses. The results for thinner films (Fig. 5a, b) are aligned with those obtained with XRD measurements. These STO films grow epitaxially with a fraction area of {001}<100> texture and the fraction of twin boundaries of 99.5 and 0.1% for 500 nm and 98.5 and 0.5% for 800 nm, respectively, whereas for the thicker sample (Fig. 5c), the surface texture is strongly deteriorated and the cube texture has not been retained. In conclusion, the orientation of the film is only preserved up to a thickness of 800 nm.

The thickness of the diffusion barrier in some applications like CC are usually between 150 and 300 nm [37]; so {001}<100> STO films obtained in this work with a thickness of 800 nm are expected to be excellent diffusion barrier.

The metal substrate is a Ni layer electroplated on a Cu clad, and consequently, at high temperature, the copper diffusion is activated while copper atoms reach the surface according to time and temperature. The copper content on nickel surface can affect the cube texture of epitaxial STO deposited or even some properties of layers deposited over STO, such as in the case of CC. In a previous research of Kashima et al. [27], with the same Cu-clad substrate used in this work, they estimated the copper concentrations on nickel layer surface at different thicknesses of nickel and different temperatures and times. For a thermal treatment of 500 °C after 2 h with a Ni layer of 0.5 μm, the content of Cu on the surface is below 1% [27]. Therefore, for Cu-clad samples used in this work with a 1 μm of Ni thickness and treated at 500 °C between 30 and 60 min, the final copper surface concentration is expected to be below 1% according to Kashima et al. [27]. For instance, it could be used for CC applications, since it can be stated that there is no effect on superconductivity if the Cu concentration rises up to 16.7% [27].

For 500 and 800 nm of (100) STO layer, the *S*_q_ values of roughness in an area of 25 × 25 μm^2^ are 5 and 7 nm, respectively, lower than the Cu-clad substrate. This low roughness is appropriate to use these (100) STO layers in a broad range of applications.

**Fig. 6:**
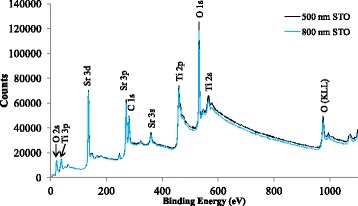
XPS spectra of STO films grown at a chamber pressure, laser frequency, and substrate temperature of 10^−4^ mbar, 10 Hz, and 500 °C, respectively, with a thickness of 500 and 800 nm

It is expected that growth of STO layers under a reducing atmosphere of a mixture of 5% H_2_ and 95% Ar are oxygen deficient. For this reason, the composition of STO layers of 500 and 800 nm were verified by means of XPS. The XPS spectra obtained are depicted in Fig. 6. As can be observed, the spectra obtained for the STO layers of 500 and 800 nm present all the expected peaks for a STO layer [38]. In both cases, the results confirm the stoichiometric composition of STO layers. At the same time, the absence of the most intense peaks for Cu and Ni, the 2p_3/2_ peak that appears at 933 and 853 eV, respectively, confirms the absence of contamination of Cu and Ni on STO surface. The data for the 2p_3/2_ peak for Cu and Ni was obtained from software MultiPak from company Physical Electronics.

Wang et al. [19] show that there is a direct relation between surface roughness and thickness of STO and superconducting properties of YBCO. They have used the PLD technique to grow high-quality STO layers on buffered ion beam-assisted deposition MgO templates and YBCO films on STO. In the present study, using Cu-clad substrate and performing the growth by PLD under reducing atmosphere and lower temperatures, the quality of STO layer obtained is comparable to those achieved by Wang et al.

## Conclusions

In summary, epitaxial STO films were grown in one step on low-cost cube-textured Cu-clad substrates using PLD under a reducing atmosphere of a mixture of 5% H_2_ and 95% Ar. SEM measurements show that (100) STO layers are continuous, dense, and smooth with a laser frequency of 10 Hz and a chamber pressure of 4 × 10^−4^ mbar, and the lowest temperature with the sharpest {001}<100> texture was obtained at 500 °C. In addition, the maximum achievable thickness of highly {001}<100> textured STO films was optimized, resulting in a STO layer of 800 nm with the best quality of texture that is preserved not only through the layer but also on the surface. It has been found that between 800 and 1000 nm of thickness, the surface texture is strongly deteriorated. AFM measurements show that (100) STO layers present a low roughness in the range of 5–7 nm. According to the XPS analysis, the layers obtained are stoichiometric. This (100) STO layer is an excellent diffusion barrier and may provide a useful template for the growth of other functional oxide layers. In the light of the results, it can be assessed that an effective method for growing highly textured (100) STO layer on low-cost metal substrates by means of PLD in the absence of oxygen has been achieved. This method opens a new path for growing other highly textured oxide layers on inexpensive metal substrates, stating the great potential of the technique.

## Abbreviations

AFM: Atomic force microscopy
EBSD: Electron backscattering diffraction
FWHM: Full width at half maximum
PLD: Pulsed laser deposition
RABiTS: Rolling-assisted biaxially textured substrate
RD: Rolling direction
SAB: Surface activated bonding
SEM: Scanning electron microscopy
SS: Stainless steel
STO: SrTiO_3_
TD: Transverse direction
XPS: X-ray photoelectron spectroscopy
XRD: X-ray diffraction
